# Single-cell disentangled representations for perturbation modeling and treatment effect estimation

**DOI:** 10.1101/2025.11.21.689783

**Published:** 2025-11-24

**Authors:** Jianle Sun, Petar Stojanov, Kun Zhang

## Abstract

Dissecting cell-state-specific changes in gene regulation following perturbations is crucial for understanding biological mechanisms. However, single-cell sequencing provides only unmatched snapshots of cells under different conditions. This destructive measurement process hinders the estimation of individualized treatment effects (ITEs), which are essential for pinpointing these heterogeneous mechanistic responses. We present scDRP, a generative framework that lever-ages disentangled representation learning to separate perturbation-dependent and perturbation-independent latent variables via a sparsity regularized ***β*** -VAE. Assuming quantile-preserving effects of perturbations conditional on confounders, scDRP performs conditional optimal transport in the latent space to infer counterfactual states and estimate ITEs. Applied to simulated and real single-cell perturbation datasets, scDRP accurately estimates treatment effects and individual counterfactual responses, revealing cell type-specific functional gene module dynamics. Specifically, it captures distinct cellular patterns under rhinovirus and cigarette-smoke extract exposures, reveals heterogeneous responses to interferon stimulation across diverse immune cell types and identified distinct functional module activation in chronic myeloid leukemia cells following CRISPR knockouts targeting different genes. scDRP also generalizes to unseen perturbation doses and combinations. Our framework provides a principled computational approach to elucidate heterogeneous causal relationships from single-cell perturbation data, promoting to a deeper understanding of cellular and molecular mechanisms.

### 1 Introduction

Understanding cell-specific heterogeneous responses to chemical or genetic perturbations is crucial to deciphering the principles of life. Advances in single-cell sequencing technologies now enable large-scale profiling of multi-omics features across numerous perturbations, offering an unprecedented opportunity to dissect gene regulatory circuitry in development and disease [1]. However, the destructive nature of sequencing experiments precludes direct comparison of a cell's state before and after perturbation-formally referred to as the individualized treatment effect (ITE). Instead, measurements consist of unmatched populations of cells under different perturbations, posing a fundamental challenge for causal inference.

To circumvent this, many current perturbation modeling frameworks, such as scGEN [2] and State [3], focus on predicting population-average responses, though successful, obscuring the treatment heterogeneity crucial in complex systems like organoids, where subpopulations respond differently. Other methods attempting individual-level prediction often resort to a branch of implausible assumptions. CINEMA-OT [4], for instance, assumes that perturbation-induced latent signals are completely independent of confounders (the cell's intrinsic properties like cell types), and that responses arise from linear combinations, neglecting complex nonlinear interactions, neglecting the complex nonlinear relationships and interactions that govern cellular responses.

In this paper, to address this challenge, we propose single-cell disentangled representations for perturbations (scDRP). Leveraging recent advances in disentangled representation learning. scDRP employs a sparsity-regularized $\beta$-VAE to separate perturbation-dependent and perturbation-independent latent variables. By assuming that, perturbations exert quantile-preserving effects on the latent variables they influence when conditioning on confounders, our framework enables the identification of ITE. Biologically, this assumption implies that a cell's intrinsic relative rank within a homogeneous subpopulation (e.g. within same cell type, with similar cell states) is preserved post-treatment. For instance, a cell that is inherently the most sensitive to the perturbation (e.g., 95th percentile) among its control peers is assumed to remain the most sensitive (95th percentile) relative to the new perturbed population, even as the entire distribution shifts. Specifically, conditional optimal transport (OT) with squared Euclidean cost, which is conceptually equivalent to quantile matching or monotone rearrangement in one-dimension [5], is performed in the disentangled latent space to generate counterfactual predictions and estimate individual causal responses (Fig.1a).

We demonstrate the strong performance of scDRP in estimating ITEs and predicting counterfactual perturbation responses across both simulated and real single-cell perturbation datasets. Biclustering analysis on the estimated ITE matrix reveals cell type-specific differences in functional gene module (FGM) responses under diverse perturbations (Fig.1b). Specifically, the re-analysis of rhinovirus and cigarette-smoke exposure data resolves distinct, fine-grained cellular patterns. Applying to more available datasets, we further uncover heterogeneous responses to interferon stimulation across diverse immune cell types, and define distinct functional module activation in chronic myeloid leukemia cells following targeted CRISPR knockouts. In addition, by interpolating within the latent and effect space, we infer the effects of unobserved perturbation doses (Fig.1c) as well as combinations of multiple perturbations (Fig.1d), highlighting the model's ability to generalize to unseen scenarios.

We believe that scDRP will provide a powerful computational tool for dissecting large-scale single-cell perturbation datasets, advancing our understanding of cellular and molecular mechanisms underlying physiological and disease processes, and paving the way for systematic *in silico* perturbation screening and rational therapeutic target discovery.

## 2 Results

### 2.1 Model overview

Inspired by VAE-based single-cell models [6], we posit a generative model for single-cell transcriptional responses to external perturbations. The model assumes that the high-dimensional gene expression profile $\mathbf{x}$ of a cell is governed by a low-dimensional latent representation (transcriptional programs) $\mathbf{z}$. We structured this latent space by decomposing it into perturbation-invariant components $\mathbf{z}_u$ and perturbation-dependent components $\mathbf{z}_d$. The invariant components $\mathbf{z}_u$ are designed to capture intrinsic cellular properties, such as cell type, which are influenced by observed confounders $\mathbf{w}$. Conversely, the dependent components $\mathbf{z}_d$ capture the cellular response, which is jointly affected by perturbations $\mathbf{a}$ and confounders $\mathbf{w}$.

To achieve this disentanglement, we enforce conditional independence between $\mathbf{z}_u$ and $\mathbf{z}_d$ given the confounders $\mathbf{w}$. We operationalize this framework using a $\beta$-Variational Autoencoder ($\beta$-VAE) [7] penalized with a Hilbert–Schmidt Conditional Independence Criterion (HSCIC) [8–10]. This HSCIC regularizer imposes the specific conditional independence structure required by our generative assumptions. This structured decomposition ensures that $\mathbf{z}_u$ and $\mathbf{z}_d$ are identifiable up to trivial transformations under some regularity conditions [11–13] (Supplementary Notes SN1).

A key assumption of our framework is that the effect of perturbation on the latent factors $\mathbf{z}_d$ is rank-preserving within defined cell subpopulations (i.e., after controlling for confounders $\mathbf{w}$ or its surrogate $\mathbf{z}_u$ since it is hard to observe all confounding). This actually corresponds to the Fréchet-Hoeffding upper bound induced by the conditional marginal distributions after controlling for confounders, specifying the conservative coupling (i.e., the minimal change assumption) that minimizes the individual causal effects [14]. This rank-preserving assumption enables the estimation of individual treatment effects (ITEs). We implement this by performing quantile matching on $\mathbf{z}_d$

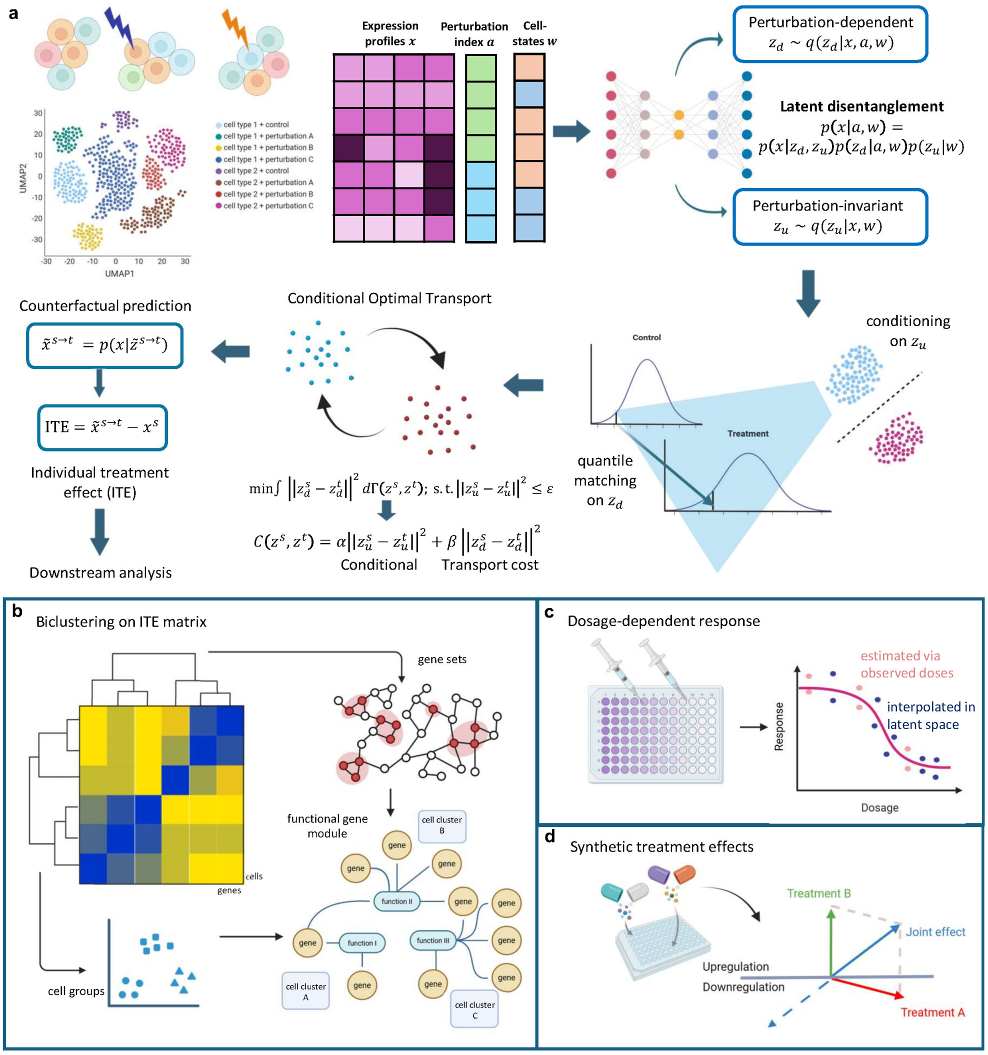

**Fig. 1** Model overview. **a.** scDRP disentangles the latent space into perturbation-dependent ($z_d$) and perturbation-independent components ($z_u$). By performing quantile matching on $z_d$ conditional on $z_u$, scDRP identifies individualized causal effects and formulates them as a conditional optimal transport problem in the latent space. **b.** Biclustering of the estimated ITE matrix reveals functional gene modules underlying perturbation responses across different conditions and cell populations. **c.** scDRP estimates effects of unseen dosages by interpolation in the latent space. **d.** scDRP infers unseen combinatorial perturbation effects by vector addition of the estimated ITEs.

between perturbed and unperturbed cells, conditional on possessing similar invariant components $\mathbf{z}_u$. This conditional quantile-matching strategy is conceptually analogous to solving a OT problem with a convex cost function $||\mathbf{z}_d^s - \mathbf{z}_d^t||_2^2$ [5] and a soft conditional $||\mathbf{z}_u^s - \mathbf{z}_u^t||_2^2$. Specifically, we seek an optimal coupling $\Gamma^*$ between the treated (target, $t$) and control (source, $s$) domains by solving

$$\Gamma^* = \arg\min_{\Gamma} \int C(\mathbf{z}^s, \mathbf{z}^t) d\Gamma(\mathbf{z}^s, \mathbf{z}^t) \in \mathbb{R}^{n\times m}, \quad (1)$$

where

$$C\left(\mathbf{z}^s, \mathbf{z}^t\right) = \underbrace{\alpha||\mathbf{z}_u^s - \mathbf{z}_u^t||_2^2}_{\text{soft conditional}} + \underbrace{\beta||\mathbf{z}_d^s - \mathbf{z}_d^t||_2^2}_{\text{transport cost}}. \quad (2)$$

Then we can predict counterfactual response and estimate ITE through barycentric projection with the optimal coupling matrix $\Gamma^*$. The details are shown in the Method section & Supplementary Notes SN2, and we illustrate the idea of conditional OT in disentangled latent space with a toy example (in Supplementary Notes SN3 & Supplementary Fig.S1-S3).

### 2.2 scDRP accurately estimate cell-specific individual treatment effects on different genes.

We first compared our approach with two representative existing methods, CINEMA-OT (COT) and scGEN. CINEMA-OT [4] assumes that the mapping from the latent space to the observed expression profiles follows a linear mixing process, and it disentangles latent components based on the strong assumption that the perturbation-related variables $z_d$ are completely independent of confounders. In contrast, scGEN [2] models the mapping from latent factors to observed variables as a nonlinear process, but it does not impose any disentanglement in the latent space; instead, it performs downstream inference based on average changes. Different from both, our model assumes that observed variables are generated as a general nonlinear function of latent variables, while simultaneously enforcing disentanglement in the latent space. Furthermore, we relax the independence assumption to require only that $z_d$ and $z_u$ are independent conditional on confounders (or their proxies, such as cell type), thereby making our framework more consistent with realistic biological settings.

We generated simulated data comprising five cell types subjected to five perturbation conditions under varying dimensionalities of $z_d$ and $z_u$. Each setting was repeated 20 times to eliminate random variation. We estimated both the ITE and CATE under two settings: with known cell-type labels (observed confounding surrogates) and with unknown cell-type labels (unobserved confounding surrogates). We evaluated the average root mean square error (RMSE) of ITE and conditional average treatment effect (CATE) across all perturbations and all cell types. As shown in Fig.2a, CINEMA-OT performs poorly on complex nonlinear data—partly because it relies on linear disentanglement, and partly due to its overly strong assumption that confounders are completely independent of the treatment. Although scGEN employs nonlinear neural networks and performs relatively well in CATE estimation through mean-shift–based adaptation instead of latent disentanglement, it performs poorly in ITE estimation.

In contrast, our method achieves consistently accurate and stable ITE estimation in both settings.

We discuss different configurations for applying optimal transport and barycentric projection in the disentangled latent space. As shown in Fig.2b, when cell types are known, the model is largely insensitive to hyperparameter choices and projection schemes, as the grouped optimal transport enables effective domain adaptation in the latent space. In contrast, when cell types are unknown, carefully balancing the weights between the conditional and transport cost components becomes more critical.

### 2.3 scDRP effectively predicts cellular responses to diverse perturbations *in silico* on real datasets.

We evaluate the effectiveness of perturbation prediction by comparing the observed real treated samples with the pseudo-treated samples generated through *in silico* counterfactual prediction from the control group. The evaluation metrics include the RMSE and Pearson correlation coefficient (PCC) of the mean expression of each gene between observed treatment group and counterfactual predictions from control group (we compare the mean since the true counterfactual outcome of control cells are not accessible in real data), as well as the maximum mean discrepancy (MMD) between the observed and predicted distributions.

To rigorously evaluate the predictive capability of scDRP, we first assessed its performance using simulated data. Regardless of the accessibility of cell-type labels, scDRP consistently demonstrated robust prediction performance, characterized by significantly lower MMD and RMSE when comparing the predicted and true treated sample distributions, alongside a notably higher PCC (Supplementary Fig.S4).

Furthermore, we proceeded to validate the counterfactual prediction performance on 4 real datasets: Kang, et.al [15], Dong, et.al [4], Hagai, et.al [16], and Adamson, et. al [17]. These datasets encompass both chemical and genetic perturbations, including experiments on pure cell lines subjected to different perturbations, as well as real tissue cells comprising multiple cell types and even multiple species, providing broad representativeness. As shown in Fig.2c (known cell-type setting) and Supplementary Fig.S5 (unknown cell-type setting), our method exhibits consistently superior performance across nearly all datasets. UMAP visualizations of counterfactual pseudo-treated samples generated from control cells (Fig.2e) further confirm this: the counterfactual distributions produced by our method almost completely overlap with the observed treatment distributions, whereas other methods exhibit substantial gaps.

The selection of hyperparameters is also a subtle issue. In real-world data, when explicit cell-type labels are used, it is better to impose no additional condition or a weaker additional condition on $\mathbf{z}_u$ (Fig.2d, Supplementary Fig.S7-S8), while without known cell-type labels, applying a moderate conditional often achieves better results (Supplementary Fig.S6). It is also worth noted that, purely matching by confounding ($\mathbf{z}_u$, i.e., setting $\beta = 0$), similar to the approach adopted by CINEMA-OT, showed very poor results.

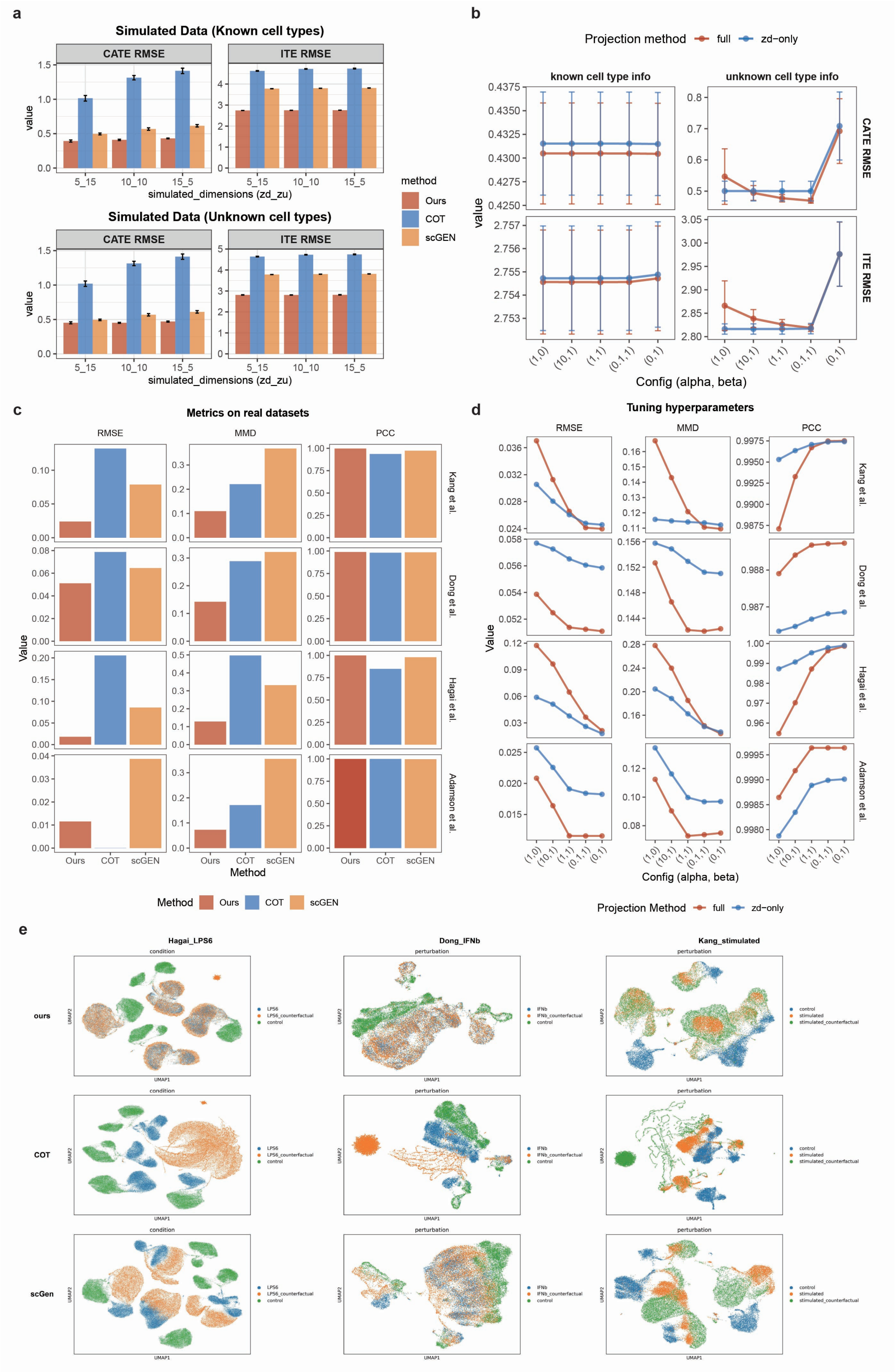

**Fig. 2** Performance of effect estimation and counterfactual prediction on simulated and real datasets. **a.** RMSE of CATE and ITE estimation on all genes under known and unknown cell type scenarios. The x-axis shows the dimension settings for $z_d$ and $z_u$ in the simulation, and the different colors correspond to different methods. We repeat each simulated setting 20 times and report the mean$\pm$1.96SE. **b.** Sensitivity analysis on hyperparameters of conditional OT on simulated data. **c.** Performance on counterfactual predictions on real single-cell data, where evaluation metrics includes RMSE and PCC of the mean expression of each gene within top 3000 highly variable genes (HVGs) between observed treatment group and counterfactual predictions from control group (we compare the mean since the true counterfactual outcome of control cells are not accessible in real data), as well as MMD between the observed and predicted distributions. All metrics are averaged across all perturbations and cell types. **d.** Sensitivity analysis on hyperparameters of conditional OT on real data. **e.** UMAP visualization of observed and counterfactual predicted cells.

### 2.4 Improved ITE estimation and biclustering of ITEs enable finer-grained capture of heterogeneous response patterns

To demonstrate the advantages of our approach, which features more accurate ITE estimation via non-linear disentanglement and conditional OT, alongside biclustering-based (rather than uni-dimensional clustering) downstream analysis for elucidating the biological heterogeneity of perturbation responses, we analyzed the same scRNA-seq dataset of rhinovirus (RV) and cigarette-smoke extract (CSE) exposure in primary human bronchial organoids as CINEMA-OT [4]. The UMAP visualization demonstrates that our more accurate ITE estimates better preserve the heterogeneity in responses to exposure across different cells (Fig.3a).

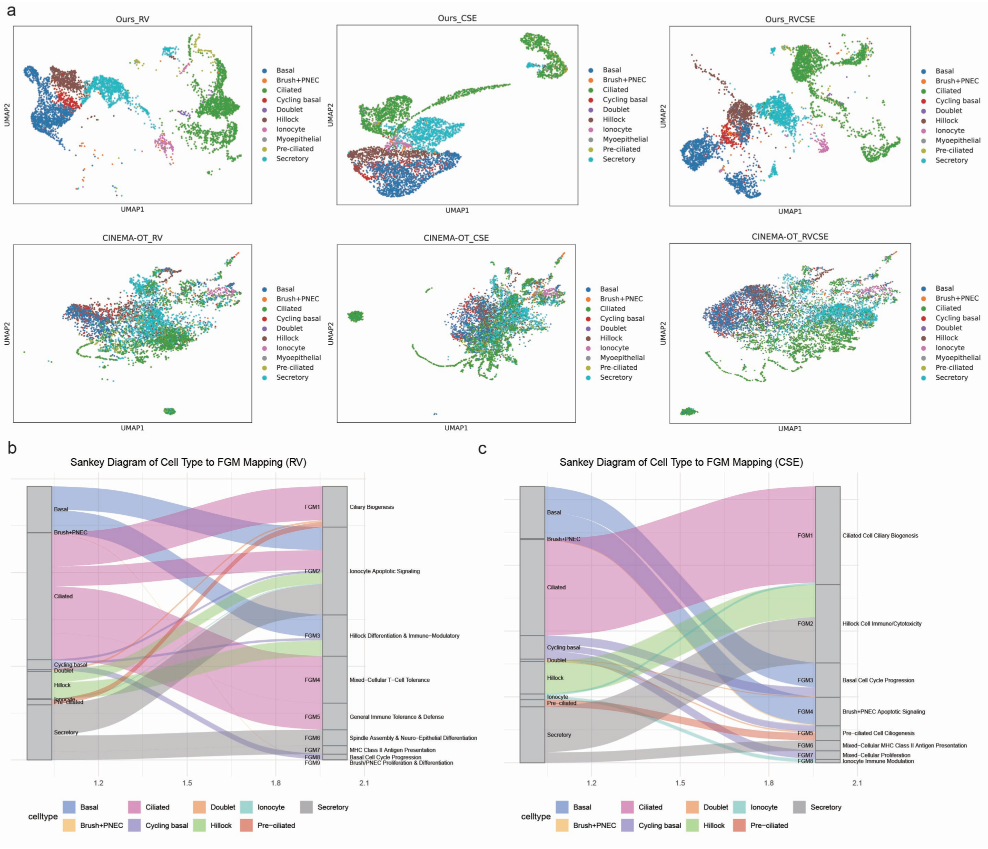

**Fig. 3** Biclustering of improved ITEs reveals fine-grained heterogeneous response patterns under RV and CSE exposures. **a.** UMAP visualization of the ITE matrix, clustering cell subgroups by their response profiles across different exposures. **b.** Sankey diagram illustrating the enrichment of FGMs within specific cell types following RV exposure. **c.** Sankey diagram illustrating the enrichment of FGMs within specific cell types following CSE exposure.

Our analysis corroborates previous findings of CINEMA-OT [4] that RV treatment induces heterogeneous, cell-type-specific effects, including the activation of apoptotic pathways and general inflammatory responses. Our more accurate ITE estimation and biclustering approach, however, resolves these effects with greater granularity by identifying distinct functional gene modules (FGMs) (Fig.3b, Supplementary Table 1, Fig.S9). For instance, we specifically localize the intrinsic apoptotic signaling pathway to the Ionocyte-dominated FGM2 module and pinpoint distinct immune profiles, such as natural killer cell mediated cytotoxicity, to the Hillock-specific FGM3 module.

More importantly, our analysis reveals major new biological effects of RV treatment not previously identified. While the prior study prominently highlighted a type I interferon signature, our ITE biclustering strategy uniquely uncovers two dominant biological programs: 1) a profound ciliary biogenesis signature (cilium organization, motile cilium assembly) localized to the Pre-ciliated/Doublet FGM1 module, and 2) a strong cell-cycle progression signature (chromosome segregation, spindle organization) enriching in both the Cycling basal (FGM8) and Brush+PNEC (FGM9) cells. These findings suggest that our approach successfully disentangles the RV ITEs into more discrete biological processes, revealing that cell-cycle and ciliogenesis perturbations [18, 19] are central, previously unrecognized components of the cell-type-specific response.

Similarly, our analysis confirms that CSE induces a heterogeneous, cell-type-specific response, with notable effects in Basal, Ciliated, and Secretory populations. Moreover, it uncovers a more complex set of biological programs than previously reported (Fig.3c, Supplementary Table 2, Fig.S10). While prior work highlighted metabolic perturbations—namely Reactive oxygen species pathway, Fatty acid metabolism, and Xenobiotic metabolism—our analysis identifies two dominant, previously unrecognized axes of the CSE ITE.

The first is a profound ciliary biogenesis failure, characterized by the overwhelming enrichment of cilium organization and cilium assembly pathways in both Ciliated (FGM1) and Pre-ciliated (FGM5) cell modules [20]. The second is a strong proliferative response, defined by chromosome segregation and cell cycle G2/M phase transition terms, which we localized to the Basal/Cycling basal compartment (FGM3) [21]. Furthermore, our approach resolves highly discrete immune and stress modules entirely missed by the prior analysis, including a potent natural killer cell mediated cytotoxicity signature in Hillock (FGM2) and Ionocyte (FGM8) cells, and an intrinsic apoptotic signaling pathway specifically within the Brush+PNEC population (FGM4) [22].

### 2.5 Biclustering on estimated ITE matrix reveals cell-type specific immune response to interferon stimulation.

We then analyzed the heterogeneous responses of different types of human peripheral blood mononuclear cells (PBMC) to interferon IFN-$\beta$ stimulation [15], which is a potent antiviral and immunomodulatory cytokine that drives a highly coordinated immune program. Visualization of the ITE matrix obtained from scDRP reveals clear clustering of different cell types, demonstrating the heterogeneous effects of IFN-$\beta$ on different immune cells, while the ITE obtained from CINEMA-OT fails to achieve this (Fig.4a). By further performing biclustering visualization of the ITE matrix obtained from scDRP reveals clear clustering of different cell types, demonstrating the heterogeneous effects of IFN on different immune cells. In contrast, the ITE obtained from CINEMA-OT fails to achieve this. on the ITE matrix stratified by cell type, followed by merging of the resulting biclusters [23], we identified 8 FGMs.

We assign each cell to the bicluster-derived FGM it belongs to, and then computed the proportion of cells associated with each FGM among each cell type. The results (Fig.4b-c) reveal distinct patterns associated between cell types and FGMs in response to IFN-$\beta$ stimulation, and we further link them with biological functions through GO enrichment analysis [24] (Fig.4d, Supplementary Table S3 & Supplementary Fig.S11). Effector cells, such as CD4$^+$ T, CD8$^+$ T, and NK cells, uniformly engage FGM3, which relates to antiviral defense and lymphocyte differentiation, confirming the primary role of IFN-$\beta$ in activating the machinery for viral clearance [25]. The CD4$^+$ T cells are also associated with FGM4, which is related to functions such as protein folding and endoplasmic reticulum stress. This suggests that the activation of T ells places enormous demands on their protein synthesis and secretory pathways, accompanied by intracellular homeostatic stress. This heightened metabolic activity is intrinsically linked to cellular homeostatic mechanisms, including the unfolded protein response (UPR) which manages endoplasmic reticulum (ER) stress, which suggests that as T cells ramp up the synthesis and secretion of cytokines and enhance antigen presentation in response to IFN stimulation, they must also engage pathways that mitigate the intracellular stress caused by the high protein load [26, 27].

Meanwhile, antigen-presenting cells (APCs), including DCs (FGM6) and B cells (FGM7), converge on MHC-II Antigen Presentation programs, highlighting a rapid mechanism to potentiate adaptive immunity. Besides, the IFN-$\beta$ stimulus links B cells with FGM8 (Immune Cell Migration) as well, indicating it also activates programs related to cell homing and localization [28]. This suggests IFN-$\beta$ prepares B cells to rapidly migrate to secondary lymphoid organs, facilitating essential T-B cell interactions necessary for antibody production [29].

Most critically, IFN-$\beta$ drives divergent functional fates within the monocyte compartment: FCGR3A$^+$ monocytes are skewed toward an activating phenotype, engaging FGM1 (MHC-I/II Presentation and T Cell Regulation), whereas the CD14$^+$ monocytes preferentially upregulate FGM2 (Apoptosis and Metabolic Negative Regulation). This functional dichotomy suggests that IFN-$\beta$ employs FGM2 as a crucial negative regulatory mechanism, possibly by limiting the lifespan and inflammatory potential of the CD14$^+$ subset to tightly control the magnitude and duration of the immune response.

#### 2.6 Chronic myeloid leukemia cells exhibit heterogeneous responses to CRISPR perturbations of different genes.

Next, we analyzed the effects of eight different single-guide RNAs (sgRNAs) mediated CRISPR perturbations on chronic myeloid leukemia (K562) cells [17]. We estimated the ITE of control-group cells under each perturbation (Fig.5a), performed biclustering of the ITEs for each perturbation separately, and then aggregated the results to obtain seven FGMs.

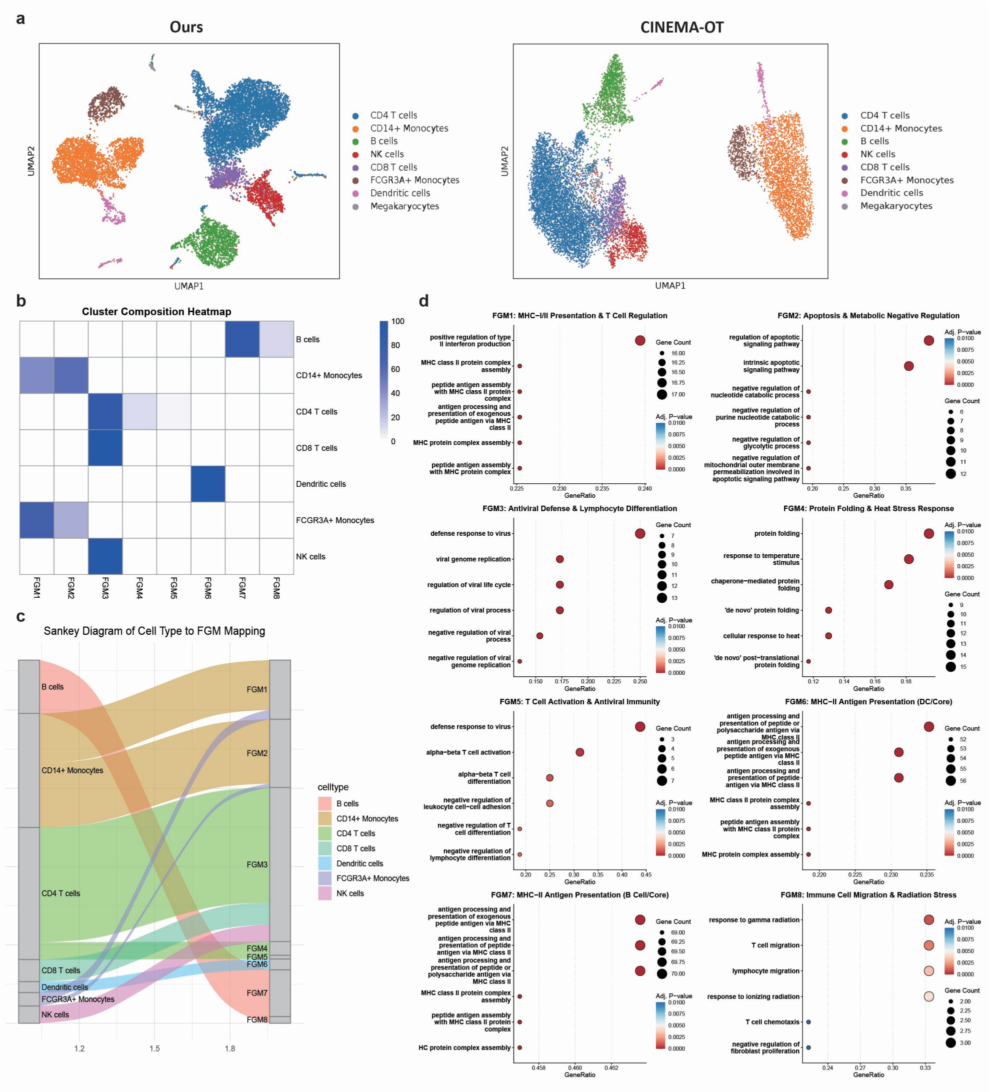

**Fig. 4** Cell-type specific immune response to interferon stimulation. **a.** UMAP visualization of ITE matrix estimated via scDRP (ours) and CINEMA-OT. **b.** Heatmap on FGM composition among each cell type. **c.** Sankey diagram of cell type to FGM mapping. **d.** GO enrichment analysis reveals functional roles of each FGM. The summary on functions of each FGM is shown in the subtitle.

Results (Fig.5b) show that FGM1 (MHC I antigen presentation and cytotoxicity) is the dominant functional module across almost all eight CRISPR perturbations, comprising 100% of the response for *UBASH3B* and over 75% for several other genes. This strongly confirms that the core response of K562 cells to genetic stress is channeled through the regulation of immune effector functions, apoptosis, and survival.

For example, miR-125b targeting of BAK1 in K562 cells has been shown to suppress apoptosis and promote proliferative signals [30].

However, specific genes show a unique divergence into other critical pathways. FGM2, which relates to cell differentiation and oxidative stress, plays a major role, particularly in the perturbations of *BAK1* (53.1%), *OSR2* (48.7%), and *KLF1* (36.5%). KLF1 has been shown to orchestrate almost all aspects of terminal erythroid differentiation in both human and murine systems [31], and in CML leukemic stem cells, shifts in oxidative stress via mitochondrial metabolism are coupled to differentiation when certain regulatory pathways are inhibited (ULK1, etc.) [32]. This suggests these genes are critical nodes linking cell death signaling to the leukemia cell's differentiation status and oxidative tolerance, where their loss may promote a dedifferentiated, proliferative state.

Furthermore, specific transcription factor perturbations reveal distinct functional consequences relevant to the myeloid lineage and immune evasion (Fig.5c, Supplementary Table S4 & Supplementary Fig.S12). CEBPE's disruption, while primarily FGM1, uniquely activates FGM7 (Phagocytosis), reflecting its key role in regulating myeloid functional maturation, especially granulocytic differentiation [33]. The co-perturbation of *CEBPE* and *RUNX1T1* is the only one to engage FGM5 (MHC I Assembly) significantly, providing a direct target to manipulate the immunogenicity of the CML cells. Lastly, the unique link of *ETS2* to FGM4 (Gas Transport and Detoxification) suggests it is a regulatory nexus controlling the leukemia cell's metabolic stability and antioxidant defense. This is consistent with broader findings that ROS regulation and metabolic reprogramming are central in myeloid leukemias, with antioxidant pathways linked to survival, immune escape, and therapy resistance [34, 35], identifying a potential vulnerability for combined metabolic and genetic targeting.

### 2.7 scDRP predicts dose-sensitive cellular response to chemical perturbations by interpolating on latent space

To assess the model's capacity to generalize to unseen conditions, we examined cellular responses across a range of chemical perturbation doses. We analysed the sciplex4 dataset [36], which profiles mammary epithelial and alveolar basal epithelial cells treated with seven compounds at multiple doses. To capture dose-specific effects, we extended the perturbation index $\mathbf{a}$ with an additional dimension encoding dose values $v$. For doses with observed cells, we inferred effects by applying optimal transport in the disentangled latent space, a procedure we term Estimation. For unobserved doses, we employ conditional Gaussian process regression to impute the mean and variance of $\mathbf{z}_d$, for unobserved dose values, and apply residual correction to ensure quantile alignment of samples before and after perturbation while keeping $\mathbf{z}_u$ unchanged. The VAE decoder is then used to generate counterfactual samples for causal effect estimation, which is a strategy we term Extrapolation (see Methods for details).

We estimated the effects of seven chemical perturbations on two cell types in the SciPlex4 dataset using two different methods. For each cell type, we selected the five genes most strongly affected and plotted the CATE–dosage curves (Fig.6a). The interpolated curves closely overlapped with those estimated at observed dose values, demonstrating the strong generalization ability of our method. In contrast, direct

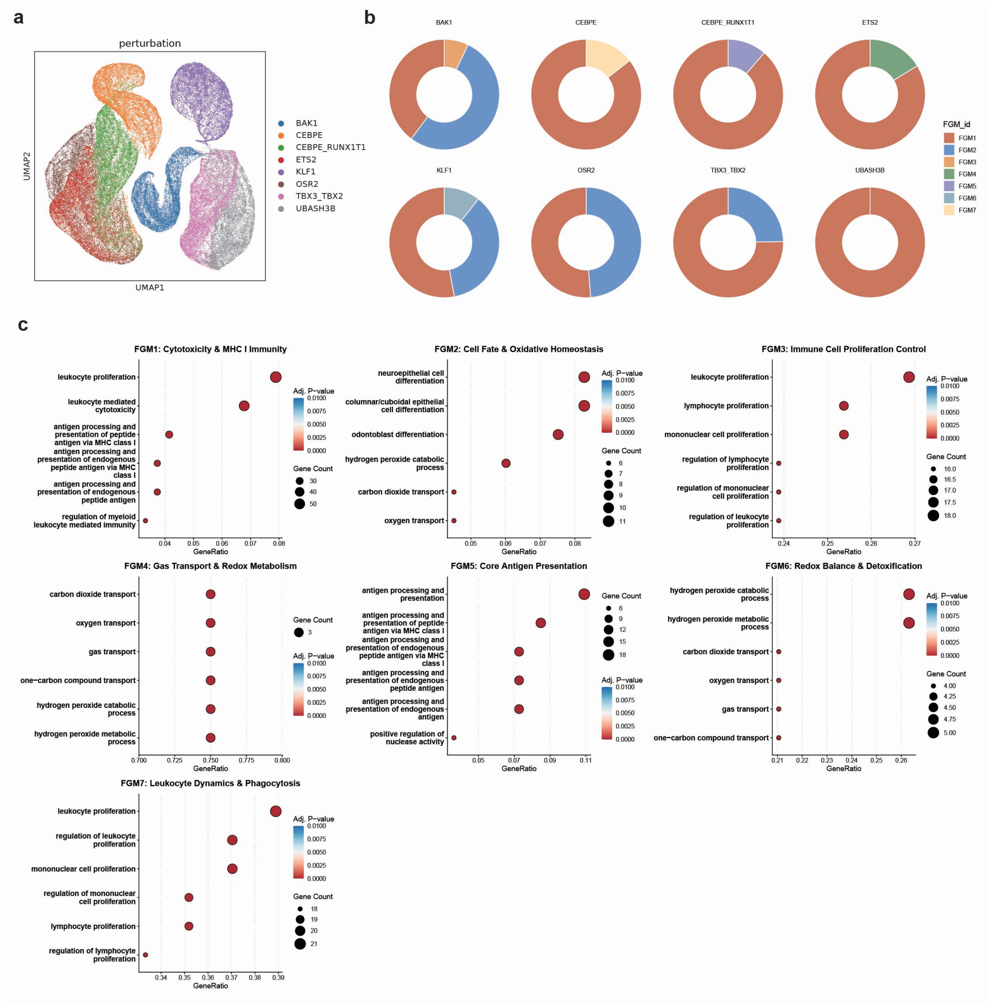

**Fig. 5** Heterogeneous responses to CRISPR perturbations. **a.** UMAP visualization of the ITE matrix for control cells under different perturbations. **b.** Proportions of FGMs under different perturbations. **c.** GO enrichment analysis reveals functional roles of each FGM. The summary on functions of each FGM is shown in the subtitle.

estimates at observed doses exhibited greater variability, as they contained more noise compared to the smoothed interpolation results obtained from the Gaussian process.

We can obtain more detailed information about perturbation effects from the dose–response curves. For example, in alveolar basal epithelial cells, Acetate stimulation leads to a dose-dependent upregulation of *AKR1B1* expression, which may be due to enhanced flux through the glucose-derived polyol pathway under high acetate conditions, increased production of NADPH to buffer oxidative stress, or activation of metabolic reprogramming that favors enzymes like *AKR1B1* [37, 38]. Indeed, *AKR1B1*

is known to mediate metabolic reprogramming and enhance stress tolerance in contexts of hyperglycemia and increased energy demand [39].

In contrast, in mammary epithelial cells, stimulation with an ACLY inhibitor exerts a sustained suppressive effect on GFRA1 expression, likely because inhibition of ACLY reduces cytosolic acetyl-CoA supply, thereby limiting lipid and protein acetylation needed for growth factor signaling and transcriptional activation; additionally, decreased ACLY activity can impair metabolic pipelines (e.g. lipogenesis, histone acetylation) [40], crucial for maintaining GFRA1 expression.

#### 2.8 scDRP helps infer synthetic effects of multiple treatments via vector composition on ITE space

To further demonstrate the model's extrapolative ability, we considered the task of inferring the synthetic effects of multiple perturbations given observations of single perturbations. We conceptualize this as a form of vector composition (see Methods). We validated this approach using a dataset [4] of human PBMCs subjected to single and combined stimulations, including IFN$\beta$ + TNF$\alpha$, IFN$\beta$ + IFN$\gamma$, and IFN$\beta$ + IL-6. We compared the absolute CATE of each gene within each cell type using ITEs estimated directly from samples subjected to combined stimulations with those calculated by vector addition of the ITEs from each single stimulation.

As shown in the Fig.6b, you can observe a very strong linear correlation, which demonstrates the effectiveness of our method in inferring combinatorial effects. At the same time, note that our method can only estimate the absolute magnitude of the combined effect, but cannot determine whether the final effect is an upregulation or downregulation of gene expression (as clearly illustrated by the absolute value function effect in the Fig.6c).

### 3 Discussions

Disentangled representation learning has recently received widespread attention to address the non-paired data challenge in single-cell omics [41]. In this study, we introduce scDRP, a framework that enables the estimation of ITEs and counterfactual responses from single-cell perturbation data. By combining disentangled representation learning with conditional optimal transport, scDRP provides a principled way to isolate the true causal impact of perturbations from confounding variation. Our results demonstrate that this formulation not only improves the accuracy of counterfactual prediction but also facilitates interpretable biological insights by linking latent dimensions to functional gene modules. Latent-space interpolation and ITE vector composition illustrates the model's capacity to generalize beyond observed experimental conditions, positioning scDRP as a versatile computational framework for identifying perturbation effects in high-dimensional single-cell systems.

We address the fundamental problem of causal inference-that only one potential outcome is observable for any given cell-through latent space decoupling and counterfactual matching. Given the complexity of biological systems and substantial technical noise, traditional linear models carry a high risk of model misspecification [42]. We therefore opted for a flexible neural network-based approach, which allows us

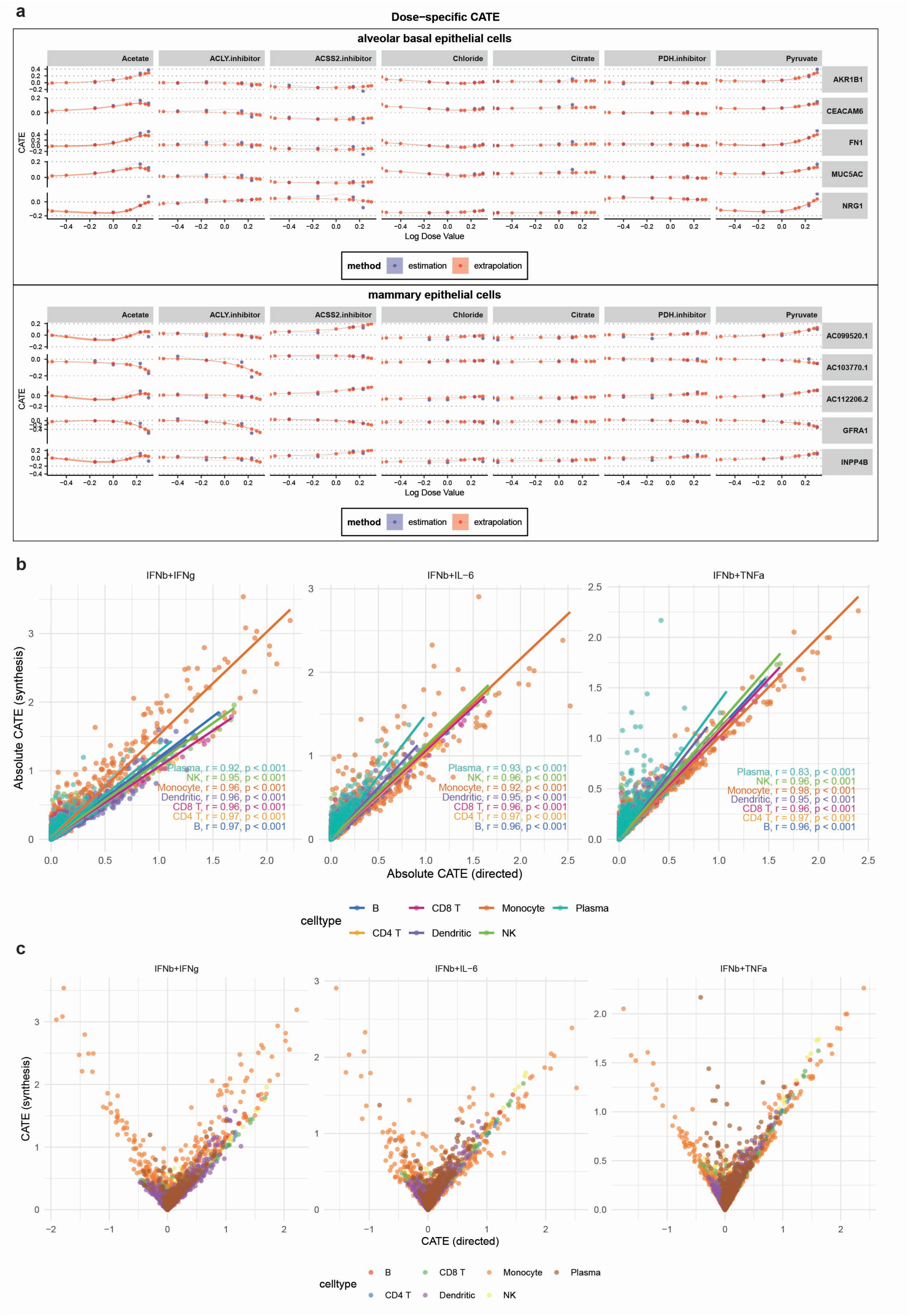

**Fig. 6** scDRP generalizes to unseen dosage values and treatment combinations. **a.** Dose-response (CATE) curves estimated via estimation (observed dosages) and extrapolation (latent interpolation) for two cell types and seven perturbations in sciplex4 dataset. **b.** Absolute CATE of combined treatments obtained via direct estimation vs synthetic inference from individual treatments for top 3000 genes. **c.** CATE of combined treatments obtained via direct estimation vs synthetic inference from individual treatments for top 3000 genes.

to effectively perform the decoupling while accommodating technical noise and batch effects. We achieve the disentanglement based by assuming that, given proxies of confounders (such as cell type), the perturbation-dependent and perturbation-invariant latent variables are independent. When cell-type information is unavailable, only partial disentanglement can be achieved. However, by incorporating the distance in $\mathbf{z}_u$ (which encodes confounding information) into the cost function of conditional OT as a soft conditional term, the model retains considerable robustness even in the absence of explicit cell-type labels.

The core idea of counterfactual matching in the latent space stems from the principle that, after controlling for confounders, a treatment may shift the overall population distribution but should not alter the relative position of each individual within the population-that is, the exogenous noise is independent of the treatment, which is commonly adopted in counterfactual inference. This actually corresponds to the Fréchet-Hoeffding upper bound on the joint distribution induced by the conditional marginal distributions of potential outcomes after controlling for confounders, specifying the conservative coupling (i.e., the minimal change assumption) that minimizes the individual causal effects [14]. We implement a soft-constrained conditional OT to jointly perform confounder control ($\mathbf{z}_u$) and quantile matching ($\mathbf{z}_d$), inspired by the equivalence between one-dimensional OT under convex loss and quantile mapping. Exploring alternative estimation strategies, such as independent residual correction or coupled per-dimension quantile matching, represents an important avenue for future work. Future extensions may also incorporate temporal or spatial modalities to further disentangle dynamic regulatory programs and cellular interactions.

While we primarily validated and analyzed scDRP on single-cell RNA-seq perturbation data in this study, the framework can be readily generalized to perturbation datasets from other omics, such as scATAC-seq [43]. We believe that as more advanced perturbation sequencing technologies are developed and more comprehensive datasets are accumulated across diverse cell and tissue types with richer perturbations, scDRP will serve as a powerful computational tool. It will help elucidate the cellular and molecular mechanisms in biological systems under perturbations at an unprecedented resolution and facilitate the discovery of novel drug targets.

## 4 Methods

### 4.1 Disentangled representations with minimal-change principle

Suppose we have the observed gene expression $\mathbf{x}$, perturbation index $\mathbf{a}$, cell type index $\mathbf{w}$ (optional), as well as some technical covariates like sequencing batch index $\mathbf{c}$ (optional). We assume observed $\mathbf{x} \in \mathbb{R}^p$ is generated from latent gene programs $\mathbf{z} \in \mathbb{R}^d$ $(d \ll p)$ and that only a subset of $\mathbf{z}$ is affected by the perturbation. Under the assumption that $\mathbf{z}_d$ are $\mathbf{z}_u$ are conditionally independent given confounding $\mathbf{w}$, we employee the following VAE architecture to learn the disentangled representations of gene expressions.

- Generative network: We assume the generating process: $p(\mathbf{x}) = p(\mathbf{z})p(\mathbf{c})p(\mathbf{x}|\mathbf{z},\mathbf{c})$, where $p(\mathbf{z}) = \prod p(\mathbf{z}_d|\mathbf{a},\mathbf{w})p(\mathbf{z}_u|\mathbf{w})$, $p(\mathbf{z}_d|\mathbf{a},\mathbf{w}) \overset{d}{=} \mathcal{N}(\boldsymbol{\mu}_{aw}, \boldsymbol{\sigma}^2_{aw}\mathbf{I}_{n_d})$ and $p(\mathbf{z}_u|\mathbf{w}) \overset{d}{=} \mathcal{N}(\boldsymbol{\mu}_w, \boldsymbol{\sigma}^2_w\mathbf{I}_{n_u})$.
  There are flexible choices to specify $p(\mathbf{x}|\mathbf{z},\mathbf{c})$ and reconstruction loss (log likelihood) accordingly. For example, (1) If the log-normalized data are provided as $\mathbf{x}$, we can use a half Gaussian distribution $|\mathcal{N}(f(\mathbf{z},\mathbf{c}),\Sigma)|$ and use mean squared error (MSE) loss as the reconstruction loss; (2) If the raw count data are provided, we may assume that $p(\mathbf{x}|\mathbf{z},\mathbf{c})$ follows a zero-inflation negative binomial (ZINB) model [6] $x_{ng} \sim \pi_g\delta_0 + (1-\pi_{ng})\mathrm{NB}(l_n\rho_{ng}, r_g) \overset{d}{=} \mathrm{ZINB}(l_n\rho_{ng}, r_g, \pi_g)$, where $l_n$ is the library size of cell $n$, $\rho_{ng}$ is the mean expression proportion of gene $g$ in cell $n$ generated by some injective functions $\rho_{ng} = f(\mathbf{z},\mathbf{c})$, $r_g$ is the dispersion factor of gene $g$, $\pi_{ng}$ is the dropout rate of gene $g$ in cell $n$, and we have $\mathcal{L}_{\text{recon}} = -\sum_n \log\{P_{\text{ZINB}}(\mathbf{z}_n; l_n\boldsymbol{\rho}_n, \mathbf{r}, \boldsymbol{\pi})\}$.
- Inference network: We use the following inference network to approximate the generative process: with encoders $q(\mathbf{z}_d|\mathbf{x},\mathbf{a},\mathbf{w})$ and $q(\mathbf{z}_u|\mathbf{x},\mathbf{w})$, and we have

$$\mathcal{L}_{\text{KL}} = \mathrm{KL}(q(\mathbf{z}_d|\mathbf{x},\mathbf{a},\mathbf{w})\|p(\mathbf{z}_{\mathbf{z}}d|\mathbf{a},\mathbf{w})) + \mathrm{KL}(q(\mathbf{z}_u|\mathbf{x},\mathbf{w})\|p(\mathbf{z}_u|\mathbf{w})).$$

To encourage disentanglement, we use $\beta$-VAE (which introduces a tunable hyperparameter $\beta$ on the KL term) [7] and impose an additional independence loss [44] on VAE ELBO by Hilbert-Schmidt conditional Independence Criterion (HSCIC) [8–10] to impose $\mathbf{z}_d \perp \mathbf{z}_u|\mathbf{w}$, i.e. $\mathcal{L}_{\text{ind}} = \mathrm{HSCIC}(\mathbf{z}_d, \mathbf{z}_u \mid \mathbf{w})$, where

$$\mathrm{HSCIC}(X, Y|Z) = \mathrm{Tr}(\mathbf{Q}G_Y\mathbf{Q}G_{\ddot{X}}),$$

which measures conditional dependence through the projection operator $\mathbf{Q} = \mathbf{I} - \mathbf{V}$ where $\mathbf{V} = (\mathbf{G}_Z + n\epsilon\mathbf{I})^{-1}\mathbf{G}_Z$ on the centered Gram matrix with the extended variable kernel $\mathbf{G}_{\ddot{X}} = \mathbf{G}_X \odot \mathbf{G}_Z$.

To encourage identifiability via sparsity constraints [11–13] and automatically select the optimal latent dimensions of $\mathbf{z}_d$ and $\mathbf{z}_u$, we introduce a gate variable $\mathbf{m}$ on $\mathbf{z}$ (i.e., use $\mathbf{m}\odot\mathbf{z}$ as the real latent factors for generating process) and then imposing hard concrete distribution on $m$ and shrinkage the latent dimension by $\mathbb{E}[||\mathbf{z}||_0] = \sum_{i=1}^{d} p_{m_i}$, where $p_{m_i}$ is the activation probability of each gate calculate via the hard concrete distribution [45].

The final loss is

$$\mathcal{L} = \mathcal{L}_{\text{recon}} + \beta\mathcal{L}_{\text{KL}} + \lambda_h\mathcal{L}_{\text{ind}} + \lambda_z\mathbb{E}||\mathbf{z}||_0.$$

### 4.2 Individual effect estimation and counterfactual prediction

In our generative model framework, the counterfactual outcomes for control group cells after treatment can be generated using the decoupled latent representations. Suppose we obtain the disentangled $\mathbf{z}_u^s \in \mathbb{R}^{n\times d_u}$, $\mathbf{z}_u^t \in \mathbb{R}^{m\times d_t}$, $\mathbf{z}_d^s \in \mathbb{R}^{n\times d_d}$, $\mathbf{z}_d^t \in \mathbb{R}^{m\times d_d}$, the perturbation-invariant latent components $\mathbf{z}_u$ will remain unchanged, while the key issue is to transfer $\mathbf{z}_d$ for samples observed in the control domain to the treatment domain. To achieve this, we transfer $\mathbf{z}_{d,i}$ from source distribution (in control group)

$\mathbf{z}_d^s$ to the target distribution (in the corresponding treatment group) $\mathbf{z}_d^t$ while conditioning on similar $z_{u,i}$. We resort to conditional rank-preserving assumption, which gives a conservative estimate that minimizes the overall ITE (minimal change principle), corresponding to the Fréchet-Hoeffding upper bound on conditional coupling $(\mathbf{z}_d^s, \mathbf{z}_d^t | \mathbf{z}_u)$ [14].

Ideally, it can be achieved by defining the cost as

$$C\left(\mathbf{z}^s, \mathbf{z}^t\right) = \mathbb{I}_{D(\mathbf{z}_u^s, \mathbf{z}_u^t)=0} C(\mathbf{z}_d^s, \mathbf{z}_d^t) + \infty \cdot \mathbb{I}_{D(\mathbf{z}_u^s, \mathbf{z}_u^t)\neq 0} C(\mathbf{z}_d^s, \mathbf{z}_d^t), \tag{3}$$

to constraint the shift of $\mathbf{z}_d$ distribution under the condition of the same $\mathbf{z}_u$. However, in reality, it would be extremely hard or impossible to find samples in two domains that are exactly matched. Even if we have some class labels as surrogates (e.g. cell types), we can do conditional transfer within each class, but the heterogeneity within each class (e.g. subclass) is still neglected. And therefore, we relax to

$$C\left(\mathbf{z}^s, \mathbf{z}^t\right) = \mathbb{I}_{D(\mathbf{z}_u^s, \mathbf{z}_u^t)\leq \varepsilon} C(\mathbf{z}_d^s, \mathbf{z}_d^t) + B \cdot \mathbb{I}_{D(\mathbf{z}_u^s, \mathbf{z}_u^t)> \varepsilon} C(\mathbf{z}_d^s, \mathbf{z}_d^t), \tag{4}$$

where $B$ is a large positive number, which is equivalent to the following cost function

$$\begin{aligned} C\left(\mathbf{z}^s, \mathbf{z}^t\right) &= \alpha D(\mathbf{z}_u^s, \mathbf{z}_u^t) + \beta C(\mathbf{z}_d^s, \mathbf{z}_d^t) \\ &= \alpha ||\mathbf{z}_u^s - \mathbf{z}_u^t||_2^2 + \beta ||\mathbf{z}_d^s - \mathbf{z}_d^t||_2^2, \end{aligned}$$

where $\alpha$ controls the strength of conditionals and $\beta$ controls the weights of transport cost, and we can get the optimal coupling matrix via optimal transport (OT) solvers

$$\Gamma^* = \arg\min_{\Gamma} \int C(\mathbf{z}^s, \mathbf{z}^t) d\Gamma(\mathbf{z}^s, \mathbf{z}^t) \in \mathbb{R}^{n\times m}. \tag{5}$$

We take both distance measure $D(\mathbf{z}_u^s, \mathbf{z}_u^t)$ and transport cost $C(\mathbf{z}_d^s, \mathbf{z}_d^t)$ as squared Euclidean distances. The rationale is twofold. First, the strict convexity of this cost ensures that in the one-dimensional case, optimal transport is equivalent to quantile matching [5], while in the multi-dimensional case it can be viewed as the sum of costs across each dimension. This makes it conceptually consistent with our assumption that perturbations correspond to rank-preserving shifts along each latent dimension. Second, since we perform disentanglement using a VAE, where the KL prior encourages the latent space to approximate a low-dimensional Gaussian distribution, adopting squared Euclidean distance as both the distance metric and the transport cost in the latent space is natural and effective.

When $\alpha = 1$ and $\beta = 0$, the procedure resembles a smoothed version of $k$NN, where matching between pre- and post-perturbation cells relies solely on the invariant component $z_u$. In contrast, when $\alpha = 0$ and $\beta = 1$, the procedure amounts to applying no conditional control, directly transferring the distribution of $z_d$ from the control group to the treatment group in a approximately quantile-preserving manner [5]. In practice, when cell types are known, we first perform domain adaptation in the latent space within each cell-type group, in which case a relatively small value of $\alpha$ can be used. By contrast, when cell types are unknown, domain adaptation is applied jointly across all samples, and a larger value of $\alpha$ is required.

Upon obtaining the optimal coupling matrix, we induce distributional shift through barycentric projection. We consider two alternative strategies: (i) performing barycentric projection over the entire latent space,

$$\tilde{\mathbf{z}}_i^{s\to t} = \frac{\sum_{j=1}^{m} \Gamma_{ij}\mathbf{z}_j^t}{\sum_{j=1}^{m} \Gamma_{ij}}, \tag{6}$$

and (ii) applying barycentric projection to $\mathbf{z}_d$ only while keeping the original $\mathbf{z}_u$ unchanged.

$$\tilde{\mathbf{z}}_i^{s\to t} = \left(\mathbf{z}_{u,i}^s, \frac{\sum_{j=1}^{m} \Gamma_{ij}\mathbf{z}_{d,j}^t}{\sum_{j=1}^{m} \Gamma_{ij}}\right). \tag{7}$$

We can then generate counterfactual samples for control group cells after treatment as $\tilde{\mathbf{x}}_i^{s\to t} \sim p(\mathbf{x}|\tilde{\mathbf{z}}_i^{s\to t}, \mathbf{c}_i)$, and the individual treatment effect (ITE) for the control cells when being treated can be estimated as

$$\hat{\boldsymbol{\tau}}_i = \mathbf{x}_i^{a=1} - \mathbf{x}_i^{a=0} = \tilde{\mathbf{x}}_i^{s\to t} - \mathbf{x}_i^s, \tag{8}$$

and the conditional average treatment effect (CATE), or more precisely conditional average treatment effect of the untreated (CATU), within a specific cell type $q$ is $\mathrm{CATE}_q = \frac{\sum_i \mathbb{I}(\mathbf{w}_i=q)\boldsymbol{\tau}_i}{\sum_i \mathbb{I}(\mathbf{w}_i=q)}$.

### 4.3 Biclustering on the ITE matrix

The ITE matrix contains information about the heterogeneity of responses to the same perturbation across different cells and genes. To uncover this heterogeneity, we employed the BCPlaid algorithm [46] (available via R package biclust, with the default parameters) to perform biclustering analysis on the estimated ITE matrix, aiming to identify subpopulations of cells and gene sets that exhibit specific response patterns.

We first perform biclustering on cell groups under different conditions (e.g., distinct cell types or perturbations), and then merge and summarize the resulting biclusters. During summarization, we first remove genes that are broadly present across all biclusters within a given condition, as these typically reflect housekeeping or background signals rather than condition-specific differences. Next, we merge biclusters within each condition, followed by merging biclusters across different conditions [23].

We merge biclusters within the same condition using the overlap score (OS) to pay more attention on exclusion, defined as

$$OS_{i,j} = \frac{e_{ij}}{\max(|g_i|, |g_j|)} \tag{9}$$

where $e_{ij}$ denotes the number of genes only appears in biclusters $i$ and $j$, and $g_i$ represents the set of genes in bicluster $i$. Bicluster pairs with $OS > 0.03$ are combined. We subsequently merge them by emphasizing the degree of overlap since biclusterings across different conditions are independent, quantified using the "intersection-over-union" (IoU) criterion

$$IoU_{i,j} = \frac{|g_i \cap g_j|}{|g_i \cup g_j|} \tag{10}$$

Pairs of biclusters with $IoU > 0.3$ are combined. Through this two-step merging procedure, we ultimately obtain functional gene modules (FGMs) that capture condition-specific biological signals.

Cells are subsequently assigned to FGMs based on their bicluster membership, allowing us to characterize changes in FGM proportions across different conditions. Furthermore, we apply the R package clusterProfiler [24] to perform enrichment analysis on these differential FGMs, thereby linking the identified modules to specific functional pathways and biological processes.

### 4.4 Unseen dose-specific responses predicted via latent space interpolation

To quantitatively describe how cells response to a particular treatment with covariates such as chemical dose values ($v$, typically log-transformed), we concatenate the perturbation dose values as one component with the one-hot encoded perturbation index to form a new perturbation index ($a$), which is then input into the model. For the dose values of cells in the observational dataset that have been subjected to the corresponding perturbation ($v_{\text{obs}}$), we can directly apply the previously mentioned method to estimate the effect size of that dose treatment on the cells (The process we refer to as Estimation).

In contrast, for dose values that have never been observed in the dataset ($v_{\text{unseen}}$), assuming $\mathbf{z}_u$ remaining unchanged, we interpolate the unknown $\mathbf{z}_d(v_{\text{unseen}})$ by using Gaussian Process (GP) regression (conditioning on $\mathbf{w}$) to predict the dose-dependent mean $\boldsymbol{\mu}_{z_d}(v)$ and standard deviation $\boldsymbol{\sigma}_{z_d}(v)$, with hyperparameters optimized by maximizing the log marginal likelihood. Personalized counterfactuals were generated based on a residual preservation principle. For each cell in the control condition, we calculated its standardized latent residual, $\boldsymbol{\epsilon}_i = (\mathbf{z}_{d,i}^{(c)} - \boldsymbol{\mu}^{(c)}) \oslash \boldsymbol{\sigma}^{(c)}$, which captures its unique deviation from the control population. Assuming this residual is conserved, the counterfactual latent state for a new dose $v_{\text{new}}$ was synthesized by transplanting this residual onto the predicted distribution: $\mathbf{z}_{d,i}^{(\text{new})} = \hat{\boldsymbol{\mu}}(v_{\text{new}}) + \boldsymbol{\epsilon}_i \otimes \hat{\boldsymbol{\sigma}}(v_{\text{new}})$. The final high-dimensional cell profile was then generated by passing the complete counterfactual vector, $[\mathbf{z}_{d,i}^{(\text{new})}, \mathbf{z}_{u,i}^{(c)}]$, through the pre-trained VAE decoder (The process we refer to as Extrapolation).

### 4.5 Synthetic effect estimation of multiple treatments through ITE vector composition

To infer the combined effects of multiple perturbations from a single-cell dataset that includes responses to different individual perturbations, we analogize the superposition of different perturbations to the addition of vectors (Fig.1d). Through decoupled learning, we can estimate the ITE for each cell $i$ in the control group after receiving perturbation $A$ ($\tau_i^A$) and perturbation $B$ ($\tau_i^B$) separately. We can then calculate the correlation coefficient $r_{AB}$ between $\tau^A$ and $\tau^B$ among all cells, which can be interpreted as the cosine of the angle between the vectors representing the effects of these two treatments. Subsequently, using the parallelogram law of vector addition and the cosine theorem, we can infer the absolute synthetic effect $\tau_i^{AB}$ when the cells simultaneously receive treatments $A$ and $B$ as

$$|\tau_i^{AB}| = \sqrt{(\tau_i^A)^2 + (\tau_i^B)^2 - 2\tau_i^A\tau_i^B r_{AB}} \tag{11}$$

Given that angles summing to 360 degrees share an identical cosine, we can only estimate the magnitude of the combined effect, while its direction (up- or down-regulation) cannot be discerned (Fig.1d). This is evident from the distinct, absolute value-like shape of the plot (Fig.6c).

### 4.6 Simulations

We simulate single-cell perturbation dataset with the following procedures. We first simulate $N$ cells in the control group, where the latent components are generated from Gaussian mixture model $z_d \sim \mathcal{N}(\mu_{at}^D, \sigma_{at}^{D^2})$ and $z_u \sim \mathcal{N}(\mu_t^I, \sigma_w^{I^2})$ with cell type labels $t$ sampled from a Dirichlet distribution $Dir(K)$. Then, for treatment group, we use the same $z_u$ and transform $z_d$ to $\mathcal{N}(\mu_{at}^D + \delta_{at}, \sigma_{at}^{D^2})$, where $\delta_{at} \sim \mathcal{N}(1, 0.5^2)$, and ensure that the ranks of the corresponding samples remain consistent before and after the shift. The mean expression of gene $g$ for each sample $\mu_{ng}$ is generated as $\mu_{ng} = \log\left\{\exp\left(f_g(z_d^{(n)}, z_u^{(n)})\right) + 1\right\}$, where $f_g(\cdot)$ is a non-linear neural network, where we impose selective masks on latent components when generating different genes to increase the sparsity. The dispersion factors $r_g$ and dropout rates $\pi_g$ are sampled to make highly expressed genes have lower Biological Coefficient of Variation (BCV, here we specify dispersion factor $r = 1/\alpha = 1/\text{BCV}^2$) and lower dropout rate, i.e., $r_g = \sqrt{\frac{5}{\mu_g/\sum_g \mu_g + 10} + 0.1 + \mathcal{N}(0, 0.01^2)}$, $\pi_g = \frac{0.4}{1+\exp\left\{0.1(\mu_g - \text{median}[\mu_g])/\sum_g \mu_g\right\}} + \mathcal{N}(0, 0.01^2)$. The gene expressions are then sampled from zero-inflated negative binomial distributions $x_{ng} \sim \pi_g\delta_0 + (1 - \pi_g)\text{NegBin}(\mu_{ng}, r_g)$. We randomly sample $N/2$ cells in control and $N/2$ cells in treatment groups to get the final observed single-cell dataset.

### 4.7 Evaluations

For the simulated data, we computed the RMSE of the estimated ITE and CATE on all genes. For the real datasets, we compared the observed treatment cells with the counterfactually generated "pseudo-treatment" samples from the control cells. We evaluated the RMSE and PCC of their mean gene expression levels within top 3000 HVGs as we cannot access the individual-level counterfactual truth. We also evaluated the MMD between the observed treatment and counterfactually predicted distributions. The MMD is calculated using top 200 HVGs, and estimated based on the empirical distributions $\hat{P}_X$ and $\hat{P}_Y$ is estimated by the V-statistic (for convenience in vectorized implementation) [47],

$$\mathrm{MMD}^2[\hat{P}_X, \hat{P}_Y] = \frac{1}{n^2}\sum_{i=1}^{n}\sum_{i'=1}^{n} k(x_i, x_{i'}) + \frac{1}{m^2}\sum_{j=1}^{m}\sum_{j'=1}^{m} k(y_j, y_{j'}) - \frac{2}{nm}\sum_{i=1}^{n}\sum_{j=1}^{m} k(x_i, y_j), \tag{12}$$

where $k(\cdot,\cdot)$ is the Gaussian (Radial Basis Function, RBF) kernel $k(x,y) = \exp\left(-\frac{\|x-y\|^2}{2\sigma^2}\right)$. The bandwidth $\sigma$ is chosen using the median heuristic, where it is set equal to the median of the Euclidean distances between all pairs of samples in the dataset. We also visualized the results using UMAP.

**Supplementary information.** Supplementary materials are available online.

**Acknowledgements.** We sincerely appreciate Dr Richard Border and Dr. Martin Jinye Zhang for their valuable comments on this work. Fig.1 is created with BioRender (https://BioRender.com).

## Declarations

- Funding: We would like to acknowledge the support from NSF Award No. 2229881, AI Institute for Societal Decision Making (AI-SDM), the National Institutes of Health (NIH) under Contract R01HL159805, and grants from Quris AI, Florin Court Capital, and MBZUAI-WIS Joint Program, and the Al Deira Causal Education project.
- Conflict of interest: No conflict of interest.
- Ethics approval and consent to participate: Not applicable.
- Consent for publication: Not applicable.
- Data availability: The Kang and Hagai datasets are available via pertpy package (http://pertpy.readthedocs.io/en/latest/index.html). The Adamson and sciplex4 datasets are available via scPerturb (https://zenodo.org/records/13350497). The Dong dataste are available from DRYAD (https://datadryad.org/dataset/doi:10.5061/dryad.4xgxd25g1).
- Code availability: Codes of scDRP are available at https://github.com/sjl-sjtu/scDRP. CINEMA-OT and scGen are implemented with Python library pertpy (http://pertpy.readthedocs.io/en/latest/index.html).
